# *Alpha-actin-2* mutations in Chinese patients with a non-syndromatic thoracic aortic aneurysm

**DOI:** 10.1186/s12881-016-0310-6

**Published:** 2016-07-18

**Authors:** Tie Ke, Meng Han, Miao Zhao, Qing Kenneth Wang, Huazhi Zhang, Yuanyuan Zhao, Xinlong Ruan, Hui Li, Chengqi Xu, Tucheng Sun

**Affiliations:** The Key Laboratory of Molecular Biophysics of Chinese Ministry of Education, College of Life Science and Technology, Huazhong University of Science and Technology, Wuhan, China; Center for Human Genome Research, Cardio-X Institute, Huazhong University of Science and Technology, Wuhan, China; Department of Molecular Cardiology, Lerner Research Institute, Cleveland Clinic, Cleveland, OH 44195 USA; Center for Cardiovascular Genetics, Cleveland Clinic, Cleveland, OH 44195 USA; Department of Cardiovascular Surgery, Union Hospital, Tongji Medical College, Huazhong University of Science and Technology, Wuhan, China

**Keywords:** Thoracic aortic aneurysm and dissection, Gene mutation, *ACTA2*, Chinese population

## Abstract

**Background:**

Aortic aneurysms and/or dissection (AADs) in the aorta are a leading cause of human morbidity and mortality. To date, data on non-syndromic thoracic AADs (TAADs) have been mainly derived from Caucasians, and the genetic basis of TAADs remains to be elucidated. In this study, we assessed gene mutations in a Chinese population with TAADs.

**Methods:**

A cohort of 68 non-syndromic familial TAAD Chinese patients was screened for the most common TAAD-causing genes (*ACTA2*, *MYH11*, *TGFBR1*, *TGFBR2*, and *SMAD3*) using high-resolution melting (HRM) analysis. Thereafter, 142 unrelated non-syndromic sporadic cases were recruited and further analyzed using HRM analysis to estimate the prevalence of disease-causing mutations in these candidate genes.

**Results:**

Two novel *ACTA2* mutations (N117I and L348R) were identified in each familial TAAD proband separately, and an additional novel *ACTA2* mutation (Y168N) was identified in one patient with sporadic TAADs. In contrast, none of the three mutations occurred in 480 control subjects. Also, no other gene mutations were identified in this cohort of Chinese TAAD patients.

**Conclusions:**

The current study identified three novel *ACTA2* mutations in Chinese TAAD patients, and these mutations represented the most predominant genes responsible for non-syndromic TAADs. In addition, HRM analysis was shown to be a sensitive and high-throughput method for screening gene mutations.

## Background

Aortic aneurysms and/or dissections (AADs) are a group of common vascular disorders characterized by cardiovascular manifestations, which include aortic dilatation*,* aneurysms, and dissection. AADs are the most common and life-threatening disorder in humans, and aortic aneurysms usually result from accumulated degenerative changes and tend to be asymptomatic until sudden dissection or rupture occurs. Therefore, the immediate mortality rate is as high as 1 %–2 %, and related medical expenditures are high. Thus, early diagnosis, prevention of sudden dissection or rupture, and treatment are critical to improve patient survival. The etiology of thoracic ADDs (TAADs) is complex and heterogeneous, and previous studies clearly showed that genetic factors contribute to the risk of the disease [1–8] in addition to environmental risk factors, such as tobacco smoking and gender or other diseases, e.g., hypertension and atherosclerosis, which also influence or modify the risk [1]. Furthermore, TAADs can occur in patients with connective tissue diseases, such as Marfan syndrome (MFS), Loeys-Dietz syndrome (LDS), and Ehlers-Danlos syndrome (EDS). A TAAD may also manifest as an isolated disease, and the majority of the non-syndromic patients have sporadic episodes. Only approximately 20 % of cases were inherited in a Mendelian fashion. The primary model of inheritance is autosomal dominant with reduced penetrance and variable expression [1, 2]. The genetic basis for non-syndromic TAADs has recently been a focus of research. Among the identified genes, *ACTA2* (localized at chromosomal 10q23-24) encodes a smooth muscle cell (SMC)-specific isoform of the contractile protein α-actin, which is the most important candidate pathogenic gene. *ACTA2* mutations are responsible for approximately 10 %–15 % of familial TAAD cases and nearly 2 %–4 % sporadic cases [3, 4]. Moreover, *TGFBR1* mutations (localized at chromosome 9q33-34) account for up to 2 % of cases, and *TGFBR2* mutations (localized at chromosome 3p24-25) account for up to 3 % of cases. *MYH11* mutations (localized at chromosome 16p12-13) account for 1 %–2 % of cases, and other gene mutations (*FBN1*, *SMAD3*, *TGFB2*, *MYLK*, *PRKG*, *MFAP5*, and *MAT2A*) have also been reported previously [5–8].

However, to date, data on non-syndromatic TAADs have been mainly derived from Caucasian populations, and mutations in these genes in Chinese TAAD patients have not been assessed so far [9, 10]. Molecular or genetic studies of TAADs could provide an insight into the mechanism underlying the pathogenesis of TAADs and benefit the development of novel strategies for early diagnosis and clinical management or prevention of sudden dissection or rupture. Toward this end, our current study analyzed the most common TAAD-causing genes (*ACTA2*, *MYH11*, *TGFBR1*, *TGFBR2*, and *SMAD3*) in 68 Chinese non-syndromic familial TAAD patients using high-resolution melting (HRM) analysis. We then confirmed the prevalence of *ACTA2* mutations in 142 unrelated cases of sporadic TAADs. The high numbers of genes and their mutations in TAADs have made the molecular characterization of this disease technically challenging. HRM analysis has been proven to be a rapid and powerful molecular biology technique for detecting genetic variations in double-stranded DNA samples [11]. Our current study utilized HRM analysis to systemically screen and identify gene mutations in a cohort of Chinese patients with non-syndromatic TAADs.

## Methods

### Study population

Patients and controls were identified and enrolled from several local medical centers in the central region of China. All patients received systemic medical assessment, and definitive diagnosis of TAADs was made on the basis of computed tomography (CT) angiography and echocardiogram. We carefully investigated specific clinical features in each patient, and the characteristics of connective tissue disorders were also identified. We excluded patients with any skeletal, muscular, dermal, or ocular defects; thus, patients with syndromic TAADs were excluded from this study. Clinicopathological data (age, gender, and other relevant medical data) were obtained from patients’ medical records and are summarized in Table 1.

**Table 1:** Clinicopathological characteristics of TAAD patients

Clinical data	Number of patients (%)
Age (years) (mean ± SD)	48.9 ± 10.8 (range 17–93)
50	106 (50.5)
50–54	50 (23.8)
55–59	28 (13.3)
≥60	26 (12.4)
Gender (male)	174 (82.9)
Risk-related diseases	
Diabetes	10 (4.8)
Hypertension	109 (51.9)
Smoker	112 (53.3)
TAAD location	
Thoracic AAD	45 (21.4)
Thoracic and abdominal AAD	165 (78.6)

Note: Thoracic AAD includes thoracic aortic dissection, thoracic aortic aneurysm, and ascending aortic aneurysmal dilatation; abdominal AAD includes abdominal aortic dissection and abdominal aortic aneurysm; thoracic and abdominal AAD includes thoracic and abdominal aortic dissection and thoracic and abdominal aortic aneurysm

### DNA extraction and gene mutation analysis

A venous blood sample (3–5 ml) from each participant was collected, and genomic DNA was extracted using the DNA Isolation Kit for Mammalian Blood (Roche Diagnostics Co., Indianapolis, IN, USA) according to the manufacturer’s protocol. After quantification, the DNA samples were subjected to HRM analysis. The polymerase chain reaction (PCR) primers were designed using online Primer 3 software to flank the coding regions of five genes (*TGFBR1*, *TGFBR2*, *ACTA2*, *MYH11*, and *SMAD3*), and the amplicon size ranged between 100 and 250 bp in length. Their map locations were verified with UCSC In-Silico PCR. Prior to HRM analysis, PCR with control samples was carried out to test the specificity of these primers (Table 2). Additional primer information is available upon request.

**Table 2:** PCR primers and HRM conditions for scanning mutations of the entire *ACTA2* coding region

Exon	Forward primer	Reverse Primer	Size (bp)	PCR Tm (°C)	HRM Tm (°C)
Exon 2	5'-tgcccaattacagctgaggct-3'	5'-tctgtgtcctgttatgttccaatca-3'	233	58	82-87
Exon 3	5'-tttgggagatgctgactcataatgtg-3'	5'-tgcatcctgagggcccaagct-3'	208	58	80-85
Exon 4	5'-catcattgtgtttctcctctgtcc-3'	5'-gaagtttccccagaccccacag-3'	197	58	82-87
Exon 5	5'-tttgtcagatgggcaccttca-3'	5'-gcgctccaaccagcttgctgtc-3'	173	58	82-87
Exon 6	5'-ccagctgccatggtgacttatc-3'	5'-cccctctcccccttatctccca-3'	235	58	82-87
Exon 7-1	5'-cacctgtgcagaccctaatgtttg-3'	5'-ggtctctgggcagcggaaac-3'	220	58	80-85
Exon 7-2	5'-ccactgccgcatcctcatcct-3'	5'-agacaatgactccccttcccag-3'	164	58	82-87
Exon 8-1	5'-acttaaggaccatggcctgtgtc-3'	5'-atcttcatggtgctgggtgctag-3'	211	58	80-85
Exon 8-2	5'-aatgtcctatcagggggcacca-3'	5'-gctgacactgctggcggc-3'	144	58	82-87
Exon 9-1	5'-ctgcactaatgatgacattaatgacc-3'	5'-ggagcaggaaagtgttttagaag-3'	210	58	82-87
Exon 9-2	5'-cgggccttccattgtccac-3'	5'-aatggtatcagtcgagtattgatcg-3'	225	58	80-85

Note: Several pairs of primers were synthesized for PCR amplification of exons 7, 8 and 9

PCR amplification was achieved in 25 μL of a PCR mixture containing 1.5 mmol/L Mg^2+^, 0.2 mmol/L dNTPs, 0.2 μmol/L of each primer, 25 ng genomic DNA, 5 μmol/L SYTO 9 green fluorescent (Invitrogen, Carlsbad, CA, USA), and 0.2 U Taq DNA polymerase (Tiangen, Beijing, China) and performed on an ABI 9700 System (Applied Biosystems, Foster City, CA, USA) with a thermal profile of 95 °C for 5 min, 40 cycles of 95 °C for 10 s, appropriate annealing temperatures for 10 s, and 72 °C for 15 s, and then a final extension at 72 °C for 10 min. PCR products were then directly screened using HRM analysis on a Rotor Gene 6000 System (Corbett Life Science, Sydney, Australia) using standard protocols with minor modifications. The optimized melting temperatures used to analyze PCR amplicons are listed in Table 2. Fluorescence data were visualized and analyzed by the Rotor Gene scanning software, and the melt curves were normalized and subsequently analyzed manually. Significant differences in the fluorescence signal from the horizontal baseline were indicative of DNA variations.

To validate the HRM results, we reanalyzed all of the samples that yielded atypical curves using conventional Sanger sequencing. DNA sequence analysis was performed using the BigDye Terminator Cycle Sequencing v3.1 kit on an ABI 3100 genetic analyzer (Applied Biosystems) as described previously [12].

### Bioinformatic analysis

To assess the deleterious effect of identified sequence variants, we used various online amino-acid substitution prediction programs, including SIFT (http://sift.jcvi.org), PolyPhen2 (http://genetics.bwh.harvard.edu/pph2), and Mutation taster (http://mutationtaster.org/) to predict whether a variant is likely to affect protein coding and functions. We then analyzed the potential effects of the mutated nucleotides on messenger RNA splicing using NetGene2 (www.cbs.dtu.dk/services/NetGene2/).

### Statistical analysis

All statistical analyses were performed using SPSS (version 17.0; SPSS, Inc., Chicago, IL, USA). A two-tailed *P* value <0.05 was considered statistically significant.

## Results

### Characteristics of the study population

In this study, we enrolled a total of 290 participants for complete clinical evaluation of TAADs and diagnosed 210 of them with unrelated non-syndromic TAADs. The mean patient age was 48.9 ± 10.8 years (range, 17–93 years), and the study population included 174 (82.9 %) men and 36 (17.1 %) women (nearly a 4.8:1 male to female ratio). The age at onset of TAADs did not differ significantly (*p* > 0.05) between genders, and 68 (32.4 %) cases had a family history of TAADs (Table 1).

### Gene mutations in TAAD patients

**Fig. 1:**
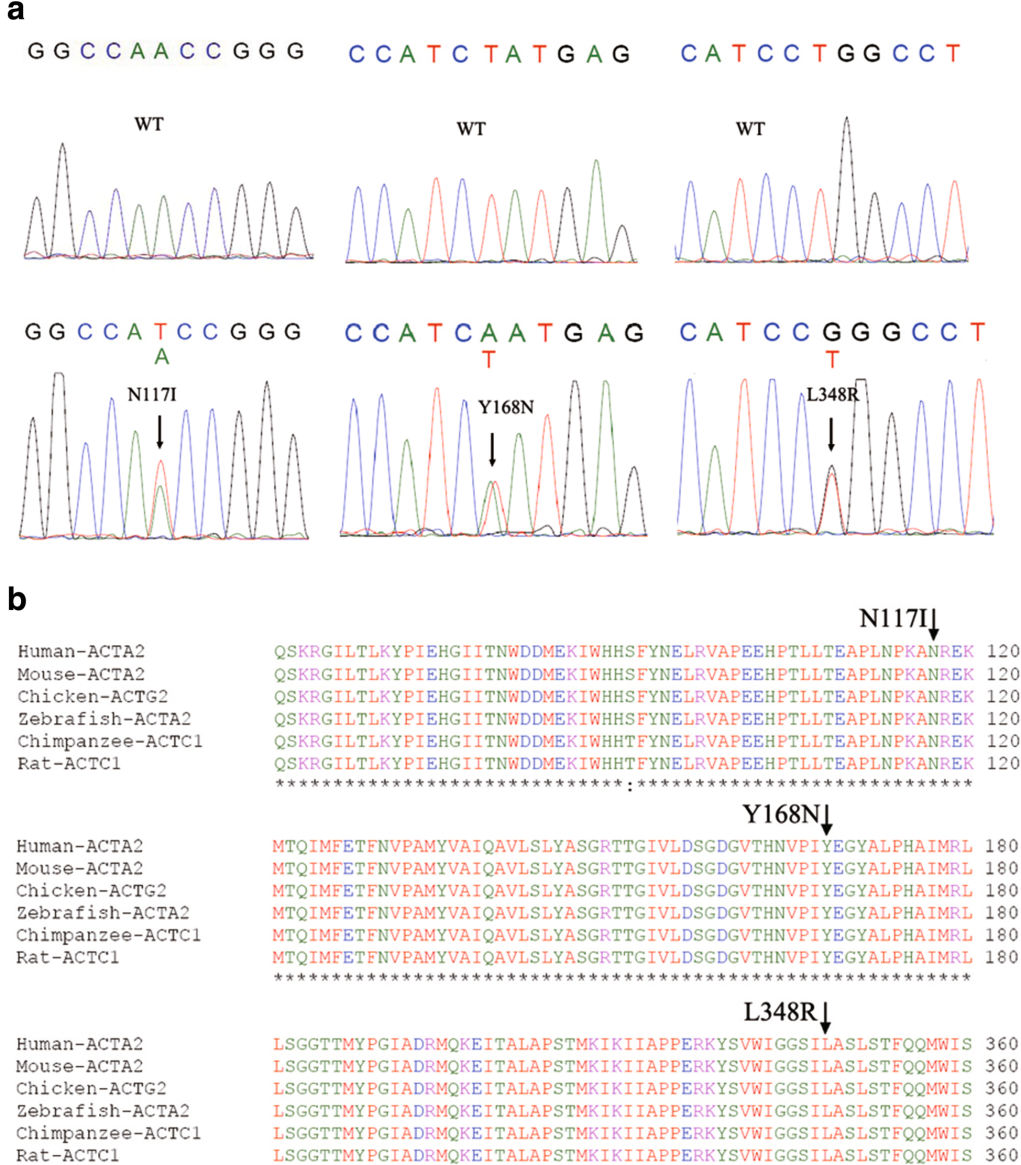
Identification of three novel *ACTA2* mutations in Chinese TAAD patients. **a** DNA sequencing data for normal controls and patients. **b** Evolutionary conservation of the ACTA2 protein sequences involving and surrounding the three mutations identified in this study. All three affected residues have been highly conserved during evolution

Sixty-eight probands with familial non-syndromatic TAADs were systematically screened for mutations of the common TAAD-causing genes (*MYH11*, *ACTA2*, *TGFBR1*, *TGFBR2* and *SMAD3*) using HRM analysis. We did not find any mutations in these four genes except *ACTA2*, and two probands (2.9 %) had a novel heterozygous missense mutation in the coding region of *ACTA2*, respectively, i.e., mutation c.825A > T (p.N117I) within exon 4 and mutation c.977 T > A (p.Y168N) within exon 6 (Fig. 1a).

We further assessed and confirmed the prevalence of disease-causing mutations of the *MYH11*, *ACTA2*, *TGFBR1*, *TGFBR2,* and *SMAD3* genes in 142 Chinese, unrelated non-syndromic sporadic TAAD patients and identified only one novel heterozygous missense mutation in one patient [1/142, 0.7 %; c.1518 T > G (L348R)] in exon 9 of the *ACTA2* gene (Fig. 1a). In contrast, no mutations within these genes were identified in the 480 healthy control individuals.

All of these three *ACTA2* mutations are absent from the dbSNP (http://www.ncbi.nlm.nih.gov/SNP), 1000 Genomes (www.1000genomes.org), and the NHLBI Exome Sequencing Project databases (http://evs.gs.washington.edu/EVS), indicating that they are novel *ACTA2* mutations.

### In silico prediction of the deleterious effects of the three identified mutations

The structure of *ACTA2* is available from the Protein database, and the mutated residues identified are mapped on the 3D protein structure. The N117 residue is found in an α-helix, whereas the Y168 residue is located outside of the regular secondary structures. The L348 residue is localized in the interior of a helix in this model (data not shown). All three mutations occurred in evolutionarily, highly conserved amino acid residues (Fig. 1b).

Furthermore, we evaluated the potential consequences in terms of altered protein function for each missense mutation using SIFT, PolyPhen-2, and Mutation taster software and found that all three mutations were likely deleterious: N117I (SIFT: affect protein function; Polyphen2: possibly damaging; Mutation taster: disease causing), Y168N (SIFT: affect protein function; Polyphen2: probably damaging; Mutation taster: disease causing), and L348R (SIFT: affect protein function; Polyphen2: possibly damaging; Mutation taster: disease causing). Taken together, our data provide strong genetic evidence that these mutations are likely pathogenic mutations in the development of TAADs.

### Clinical features of mutation-positive patients

The clinical features of the patients carrying an *ACTA2* mutation are summarized in Table 3.

**Table 3:** Characteristics of individuals with *ACTA2* mutations

Family patient	Age (y)/sex	Exon	Nucleotide	Amino acid change	Inheritance	Clinical characteristics
			change (GRCh37/hg19)			
Family 1						
I:1	44/male	4	chr10:90703573 T > A	p.N117I	Familial	TAAD, stroke
			cDNA.825A > T			
II:1	39/male	4	chr10:90703573 T > A	P.N117I	Familial	TAAD, AAAD,hypertension,
			cDNA.825A > T			
Family 2						
I:2	56/female	6	chr10:90695071A > C	p.L348R	Familial	TAAD
			cDNA.1518 T > G			
II:1	38/male	6	chr10:90695071A > C	p.L348R	Familial	TAAD, AAAD
			cDNA.1518 T > G			
III:1	12/male	6	chr10:90695071A > C	p.L348R	Familial	no symptom
			cDNA.1518 T > G			
Family 3						
II:1	31/male	9	chr10:90701100A > T	p.Y168N	*De novo*	TAAD,BAV, AS
			cDNA.977 T > A			

*TAAD* thoracic aortic aneurysms and/or dissection, *AAAD* abdominal aortic aneurysms and/or dissection, *BAV* bicuspid aortic valve, *AS* aortic stenosis. Roman numerals indicate the generation (I, II or III) and Arabic numbers (1, 2, or 3) indicate the birth order of siblings

#### Patient 1 and family

**Fig. 2:**
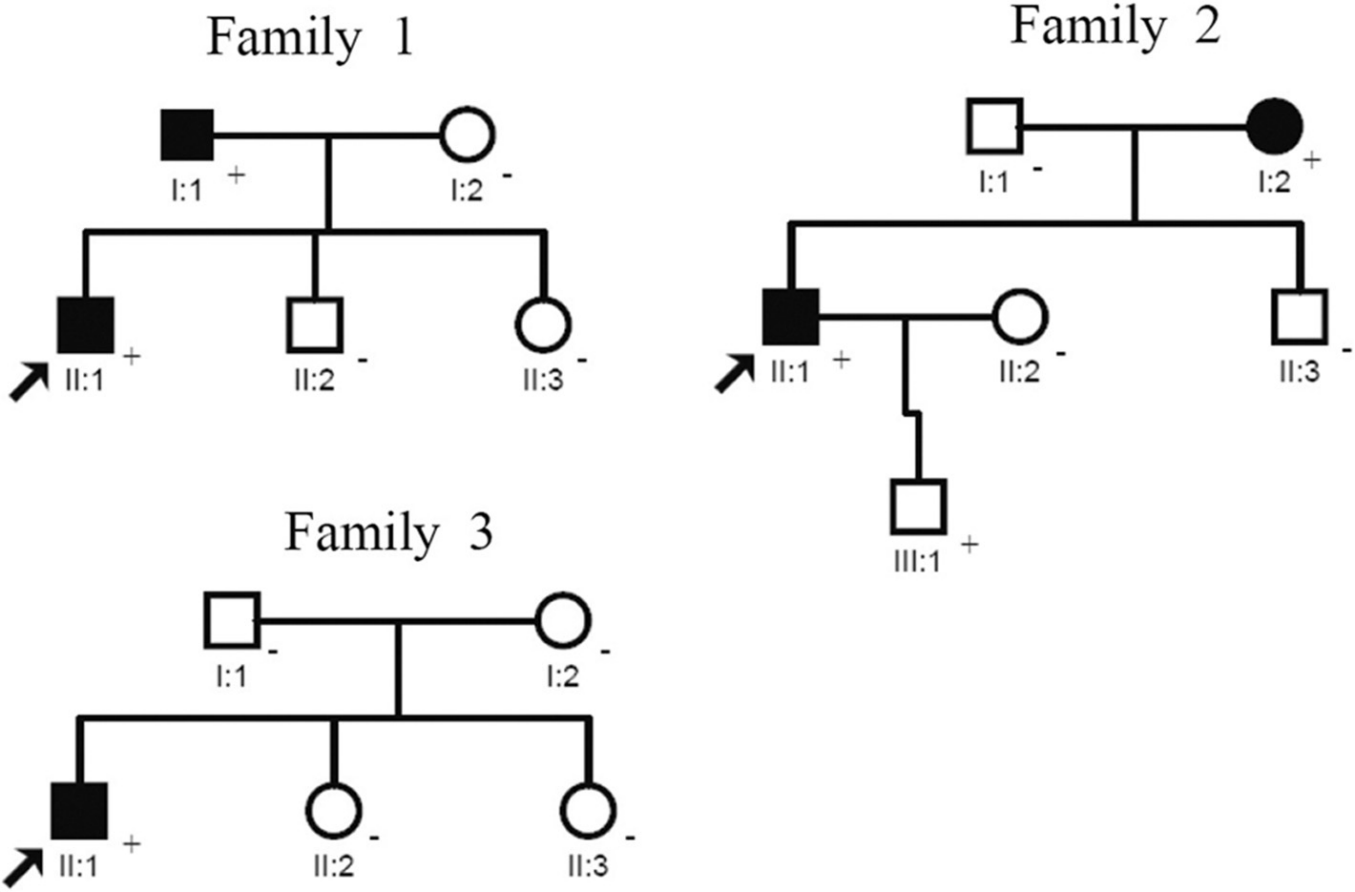
Pedigree of Chinese families with *ACTA2* mutations. Affected males and females are indicated by filled squares and circles, respectively, and normal individuals are shown as open symbols. Arrow: the proband, +: mutation present, −: mutation absent. Roman numerals indicate the generation (I, II or III) and Arabic numbers (1, 2, or 3) indicate the birth order of siblings

A 39-year-old male proband presented with acute chest pain. His medical history included 8 years of hypertension and a type A dissection originating from the aortic root and stretching to the abdominal aorta. The primary entry was located on the aortic sinotubular junction. The maximal diameter of the ascending aorta was 6.0 cm, and some small ruptures were found on the aortic arch and descending aorta. At 27 h after hospital admission, the patient underwent emergency surgery of the Bentall procedure with total arch replacement and a frozen elephant trunk. His recovery was uneventful. A missense mutation N117I was detected in this proband, and this mutation occurred in the affected patient’s father also and not in other family members (Fig. 2).

#### Patient 2 and family

A 38-year-old male proband was diagnosed with an acute type B dissection. CT angiography showed that the dissection originated from the ostia of the left subclavian artery (LSA) and stretched to the right common iliac artery. The primary entry was located on the lesser curvature of the descending aortic arch. The bilateral renal arteries, celiac trunk, and superior mesenteric artery were supplied by the true lumen of the abdominal aorta. The patient underwent endovascular repair with a covered-stent graft from Medtronic (30 × 160 mm) without any complications. The mutation L348R was identified in this TAAD patient, his affected mother, and his unaffected son, but not in other family members who underwent genetic testing (Fig. 2 and Table 3).

#### Patient 3 and family

A 31-year-old male patient was admitted to the clinic for assessment of the cause of a cardiac murmur after birth and intermittent dizziness for 3 years. Routine ultrasonic cardiogram revealed a bicuspid aortic valve with severe stenosis and aneurysmal dilatation of the ascending aorta. CT angiography showed that maximal diameter of the ascending aorta was 5.7 cm, and the three main branches of the aortic arch were normal. The patient underwent a successful Bentall surgery. A missense mutation Y168N was identified in this patient, but this mutation was absent in both parents. The paternal relationship was confirmed by genotyping and linkage analysis of five highly polymorphic microsatellite markers (data not shown). These results indicate that the Y168N mutation is a *de novo* mutation (Fig. 2 and Table 3).

## Discussion

Multiple genetic causes of non-syndromic TAADs have been evaluated and identified in patients from Europe, North America, and Japan, but little is known about such causes in Chinese TAAD patients. In the current study, we examined 210 Chinese patients with non-syndromic TAADs by screening for mutations in the most frequent TAAD-causing genes, but we failed to detect the mutations of *MYH11*, *TGFBR1*, *TGFBR2*, and *SMAD3*. We did successfully identify three *de novo ACTA2* mutations in our cohort of patients. The data provide direct evidence that mutations of *ACTA2* are likely the major cause of non-syndromic TAADs in Chinese patients.

Because the molecular mechanisms of TAAD development involve multiple genetic factors, molecular analysis of TAAD patients is challenging. Whole exome sequencing is indeed increasingly being used to investigate the genetic basis of TAADs [13, 14], but to date, most of the mutational screening in patients has been performed by direct DNA sequencing. However, both conventional method for large-scale detection of mutations and whole exome sequencing are expensive and technically time-consuming. Thus, HRM analysis has been successfully used to overcome these limitations and constitutes a detection method with a nearly 100 % detection accuracy [15]. In our current study, we developed a fast genetic HRM screening strategy to identify gene mutations in TAAD patients, and to the best of knowledge, this is the first study to apply HRM technology for scanning TAAD-associated genes in a large cohort of patients. Our results demonstrate that HRM is a sensitive and high-throughput method for the identification of TAAD-causing mutations.

Thus, our current study was able to detect *ACT2A2* mutations in 2.9 % (2/68) of familial cases and 0.7 % (1/142) of sporadic cases, rates which are significantly lower than those previously reported for *ACTA2* mutations in North America, Europe, and Japan (approximately 10 %–15 % of familial TAAD cases and 2 %–4 % of sporadic cases) [3–21]. There are three possible reasons for these discrepancies. First, these differences may be due to unidentified *ACTA2* mutations within noncoding regulatory sequences of *ACTA2* or the intron region or the presence of large insertion/deletions of *ACTA2* locus that may contribute to TAAD risk. Secondly, it is possible that the prevalence of *ACTA2* mutations in the Chinese population was underestimated due to our limited sample size. Thirdly, the differences in the detection rate of TAAD-causing mutations in *ACTA2* may be racially related. For example, it has been reported that the frequency of causal mutations or genomic variants (i.e., population-specific single nucleotide polymorphism) in some cardiovascular diseases, such as hypertrophic cardiomyopathy, long QT syndrome and coronary artery disease, are obviously different between Caucasian and Chinese populations [22–24]. In addition, although previous studies identified a higher rate of *ACTA2* mutations, the cohorts may have been selected through a special medical or genetic program in those studies. Similar to the findings in our study, Bee et al. reported a significantly lower rate of *ACTA2* mutation (3 %) in 100 probands [20].

Since the first TAAD-causing *ACTA2* mutation was identified in 2007, more than 20 different *ACTA2* mutations have been identified and are scattered throughout the whole coding region of *ACTA2* [3, 4, 16–21]. Specific *ACTA2* mutations are associated with particular forms of vascular involvement. In our current study, we found three novel *ACTA2* mutations, and the 117-arginine residue was affected by mutation of the threonine residue identified previously [16]. Thus, our current data suggest that Arg117 can also be mutated to an isoleucine residue. Notably, although the clinical manifestations of patients (I:1 and II:1) with the *ACTA2* R117I mutation in family 1 are variable, a TAAD was the first and only symptom that has been presented by this proband, whereas another mutation carrier (I:1) had a stroke that occurred in middle-age and the proceeded to have a TAAD. Although genetic alteration has been previously reported at the location R117, stroke was found to occur in Caucasian individuals with the p.R117T mutation [16]. We observed stroke in the setting of the mutation altering R117 in our Chinese population as well, and this finding further supports that *ACTA2* mutation may be responsible for stroke in TAAD patients. However, to date, although the genotype–phenotype correlation has not been fully documented for *ACTA2*-related TAADs, it is suggested that the majority of aortic aneurysms or dilatation in patients with an *ACTA2* mutation may result in dissections at diameters below classic surgical thresholds (aortic root diameter <50 mm) [17]. In our current study, the aortic diameter of proband II:1 (family 1) was 6.0 cm at the time of dissection. In family 3, the patient II:1 had a maximal ascending aortic diameter of 5.7 cm, and aortic aneurysmal dilatation was associated with bicuspid aortic valve and aortic stenosis. All these inter- and intra-family phenotypic variations may suggest environmental or genetic factors play important roles in the development of TAADs and *ACTA2*-associated vascular diseases.

The molecular consequences of the three identified novel *ACTA2* mutations are still unknown, and most *ACTA2* mutations identified so far are missense mutations, which act via a dominant negative mechanism. Thus, we speculate that these three novel mutations may act similarly to other heterozygous *ACTA2* mutations, and such substitutions may disrupt actin filament assembly or stability, impair contraction of SMCs, lead to vascular remodeling, and ultimately contribute to an increased aneurysm and dissection susceptibility [3, 25]. Furthermore, recently, enhanced transforming growth factor beta (TGF-β) signaling with downstream canonical pSmad2 upregulation has been described in TAADs, and regulation of TGF-β signaling and SMC function and proliferation are all involved in the pathogenesis of TAADs [21]. Thus, further studies are needed to better understand the pathogenesis of TAADs in Chinese patients.

Our current study has several potential limitations. For example, we analyzed only *ACTA2*, *MYH11*, *TGFBR1*, *TGFBR2,* and *SMAD3* in the familial and sporadic TAAD cases, but not other genes, such as *FBN1*, *SMAD3*, *TGFB2*, *MYLK*, *PRKG*, *MFAP5*, and *MAT2A*, which may be altered (although potentially less frequently) in non-syndromic TAAD in the Chinese population. Furthermore, our study lacked *in vitro* functional confirmation tests to illustrate the functional changes resulting from this gene mutation, and thus, further research is needed to better understand the pathogenesis of non-syndromic TAAD.

## Conclusion

In conclusion, the current study evaluated *ACTA2* mutations in Chinese TAAD patients and identified three novel *ACTA2* mutations that may have contributed to the development of TAADs in these patients. Thus, detection of *ACTA2* mutations could be useful for molecular diagnosis and genetic counseling for Chinese TAAD patients. Moreover, the current study demonstrated that HRM is a useful and high-throughput technique for detecting gene mutations in clinical samples.

## Highlights

- HRM analysis is a useful technique for screening gene mutations in TAAD patients.
- Three novel *ACTA2* mutations were identified in Chinese non-syndromatic TAAD patients.
- Racial/ethnic traits may explain the low rate of *ACTA2* mutations in Chinese patients.
